# *Porphyromonas gingivalis* W83 traffics via ICAM1 in microvascular endothelial cells and alters capillary organization *in vivo*

**DOI:** 10.1080/20002297.2020.1742528

**Published:** 2020-03-26

**Authors:** L. Reyes, H. Getachew, W.A. Dunn, A. Progulske-Fox

**Affiliations:** a Department of Pathobiological Sciences, University of Wisconsin - Madison, School of Veterinary Medicine, Madison, WI, USA; b Department of Oral Biology, College of Dentistry, Center for Molecular Microbiology University of Florida, Gainesville, FL, USA; c Department of Anatomy and Cell Biology, University of Florida, Gainesville, FL, USA

**Keywords:** Periodontitis, microvascular endothelium, *Porphyromonas gingivalis*, ICAM-1

## Abstract

**Objective:** Microvascular dysfunction is a feature of periodontal disease. *P. gingivalis*, one of the most common oral bacteria present in gingival tissue biofilms, has also been identified in the gingival capillaries of patients with chronic periodontitis. We sought to determine the effect of *P. gingivalis* W83 infection on microvascular endothelium *in vivo* and *in vitro*.

**Methods and Results:** Interdental papillae of rats with *P. gingivalis*-induced alveolar bone loss had a more dilated and denser subepithelial capillary network than uninfected controls. *P. gingivalis* W83 was detected in the epithelial layers, the subepithelial connective tissue matrix, and subgingival capillaries. *P. gingivalis* invaded human dermal microvascular endothelial cells (HD-MVECS) and persisted up termination (24 h). Colocalization analysis at 2.5, 6, and 24 h post-inoculation showed that 79–88% of internalized bacteria were in ICAM-1 positive endosomes, and 10–39% were in Rab5, Rab7, or LAMP1 positive compartments, but never in autophagosomes. Antibody-based blockade of ICAM-1 significantly reduced W83 invasion in HD-MVECS. *P. gingivalis* infected HD-MVECS were unable to form vascular networks in Matrigel.

**Conclusions:** *P. gingivalis* perturbs microvascular endothelial function and invasion of these cells via ICAM-1 may be important for microbial persistence within tissues.

## Introduction

Periodontitis is commonly defined as the irreversible destruction of the supporting periodontal tissues with alveolar bone loss and pocket formation [1,2]. Beneath the gingival crevicular epithelium is a capillary network that also becomes dysfunctional during periodontitis in that the subgingival capillary network undergoes extensive remodeling in which blood vessels become dilated and tortuous [2,3]. This results in a minor increase in permeability due to larger gap junctions between cells [4]. It is thought that the driver of vascular remodeling during periodontal disease is the protracted exposure of endothelial cells to pro-inflammatory mediators and bacterial toxins that may promote cell death [2].

There is long-standing evidence that bacteria invade the gingival tissues of patients with periodontitis. Electron and light microscopic studies of gingival biopsies from periodontitis patients have demonstrated the presence of fusiform, coccobacilli, and spirochetes within the gingival oral epithelium and adjacent connective tissue [5–8]. In particular, *Actinobacillus actinomycetemcomitans, Fusobacterium nucleatum, Prevotella intermedia, Treponema denticola*, and *Porphyromonas gingivalis* have been detected in gingival epithelial cells and the subgingival connective tissue matrix [1,8,9]. More recently, *P. gingivalis* and *Tannerella forsythia* have been identified in the capillaries of gingival and subgingival tissue specimens obtained from patients with chronic periodontitis [10], confirming that microbial invasion of the subgingival capillary network also occurs during disease. A notable feature of this study was that *P. gingivalis* and *T. forsythia* were observed in the capillaries of patients with chronic disease but not aggressive periodontitis. Furthermore, intracellular bacteria when present in capillary endothelium was always accompanied by intracellular colonization of stromal inflammatory cells and extracellular colonization of the gingival tissue matrix [10].

Endothelial cells exhibit extensive structural and functional heterogeneity that is driven by the local tissue microenvironment [11]. Although endothelial cells undergo phenotypic drift when removed from their native environment, they still retain some tissue-specific characteristics [11]. For example, comparative studies between gingival microvascular endothelial cells, dermal microvascular endothelial cells (HD-MVECS), and human umbilical vein endothelial cells (HUVECS) show some similarities and distinctive features among these cell types. All three cell-types express plasminogen activators, plasminogen activator inhibitor-1, form a tubular network in Matrigel, and show increased expression of cell adhesion molecules in response to bacterial LPS or cytokines [12–15]. However, HUVECS display different cell adhesion molecules and cytokine expression profiles compared to gingival and dermal microvascular endothelium [13,16,17]. Therefore, HD-MVECS likely more accurately model microbial/endothelial interactions within the gingival capillary network.

Since *P. gingivalis* invades and perturbs endothelial cells from large vessels [18–21], and is one of the most common invaders of gingival tissue [8–10], we sought to determine the impact of *P. gingivalis* infection on microvascular endothelium *in vivo* and *in vitro*. We found that rats experimentally infected with *P. gingivalis* developed a denser more dilated subgingival capillary network consistent with periodontal disease. *P. gingivalis* infection of HD-MVECS also disrupted their ability to form capillary-like networks in Matrigel without killing host cells. Moreover, we report here that *P. gingivalis* effectively invades HD-MVECS via Intercellular Adhesion Molecule 1 (ICAM-1) mediated endocytosis.

## Materials and methods

### Bacterial strain and culture conditions

We used a working stock of *P. gingivalis* strain W83 that invades human coronary artery endothelial cells and primarily traffics through the autophagic pathway in these cells [22]. Bacteria were cultured as previously described [22]. Briefly, bacteria were maintained on blood agar plates (5% sheep blood, Quad-Five, Ryegate, MT, USA) supplemented with vitamin K1, hemin, yeast extract, L-cysteine hydrochloride (sBAP) and gentamicin (50 µg/ml; Sigma-Aldrich, St. Louis, MO, USA). Inoculates were prepared from stationary phase cultures grown in supplemented tryptic broth (sTSB) without antibiotics. All cultures were incubated at 37°C in an anaerobic chamber (5% CO2, 10% H_2_, and 85% N_2_) (Coy Products, Ann Arbor, MI, USA). Bacterial concentrations of all inoculates were initially determined by optical density readings taken at 550 nm, that had been confirmed by culture. For all infection experiments, bacterial suspensions were diluted in cell culture media to achieve an MOI of 100. All procedures were done in accordance with University of Florida and University of Wisconsin Environmental Health and Safety policies.

### Animal studies

All procedures were conducted with approval from the University of Wisconsin Institutional Animal Care and Use Committees. All experiments used specific pathogen free Sprague Dawley rats (Charles River International Laboratories, Inc., Kingston, NY). Animals were housed in the same room, fed sterile food and water, and always handled within a biosafety cabinet. In all experiments, control animals were always handled before infected animals.

An oral inoculation protocol was used to establish a chronic periodontal infection in rats [23]. Six to 8-week-old female SD rats first received kanamycin (20 mg) and ampicillin (20 mg) daily for 4 days in the drinking water to reduce the number of commensal bacteria. The oral cavity was then swabbed with 0.12% chlorhexidine gluconate (Peridex: 3M ESPE Dental Products, St Paul, MN, USA) mouth rinse to inhibit the endogenous organisms and to promote subsequent colonization with *P. gingivalis* strain W83. Rats were switched back to antibiotic free water and rested for 3 days to allow clearance of antibiotics from their system before beginning the inoculation phase of the study. Rats were randomly assigned to control or *P. gingivalis* W83 groups. *P gingivalis* was mixed in sterile 2% (w/v) low viscosity carboxymethylcellulose (CMC; Sigma, St Louis, MO, USA) at a concentration of 1 × 10^10^ bacteria CFU per ml. Each animal received an oral inoculation containing 1 × 10^9^ CFU for 4 consecutive days per week on 6 alternate weeks totaling 24 inoculations over a 12-week period. Control animals received sterile 2% CMC. During the inoculation period, animals were fed a gamma-irradiated powdered rodent diet (Teklad Global 18% protein rodent diet, Envigo, Madison, WI) in order to minimize disruption of bacterial plaque. At the end of the inoculation period, rats were switched back to the same diet in a pelleted form and rested for 1 week [23]. Because these animals were part of a pregnancy study they were euthanized at gestation day 18, which resulted in animals being euthanized between 4 and 5 months after the first inoculation.

### Histology, morphometric analysis, and in situ staining for *P. gingivalis*

Both the mandible and maxilla were collected for assessment of alveolar bone loss and gingivitis, respectively. Mandibles were disarticulated from the skull and most of the tissue was removed by dissection. Remaining tissue was removed by Dermestid beetles. Rat mandibles were then immersed in a 3% (v/v) hydrogen peroxide solution overnight, washed with deionized water and air dried. In order to delineate the cemento-enamel junction (CEJ), jaws were stained with a 0.1% (W/V) methylene blue for 1 min, washed and air dried. Specimens were coded to prevent bias and calibrated images of both the buccal and lingual side of each jaw were captured with an EOS 650D RebelT41 camera (Canon, USA, Inc., Long Island, NY). Morphometric measurements were performed blinded to treatment. Calibrated digital images were analyzed with Image J 1.50b analysis software (Rasband, National Institutes of Health, USA). Area measurements (mm^2^) of both the lingual and buccal surface of each mandible were determined by using the freehand tool to manually trace the surface perimeter of the cemento-enamel junction (CEJ) and alveolar bone crest (ABC) as shown in supplement file, Figure S1(a). All four measurements from each animal were added and reported as a single data point.

Maxillae were fixed overnight in Neutral Buffered Formalin10% (EMD Millipore Corp., Billerica, MD), then decalcified with 10% EDTA for 8 weekly exchanges. Processed tissues were embedded in paraffin. Histologic evaluation was performed blinded to treatment on Hematoxylin and Eosin stained sections. Calibrated images of the interdental papillae from each specimen were taken with an EVOS FL Auto Cell Imaging System (Life Technologies, Grand Island, NY). For morphometric analysis, digital images were then analyzed with Image J 1.50b analysis software (Rasband, National Institutes of Health, USA). The interdental papillae were considered the region of interest (ROI), and their area was determined by a manual tracing that encompassed the gingival epithelium and underlying connective tissue matrix up to the cemento-enamel junction (supplement file, Figure S1(c)). Vascular density measurements were determined by manually counting all vessels within the ROI for all papillae present within the specimen. Final determinations were made by adding all vessel counts from multiple sites and dividing by the total ROI area (sum area measurements of all papillae). Capillary dilation was measured by determining the luminal area of each vessel within the ROI, which was also done by manual tracing (Supplement file, Figure S1(d)). All vessel lumen area measurements from each animal were then divided by the total ROI area and reported as a percent.

*In situ* detection of *P. gingivalis* proteins within rat interdental papillae was done with a rabbit polyclonal antibody specific to whole cell *P. gingivalis* strain W83 as previously described [23,24]. Briefly, formalin-fixed paraffin specimens underwent antigen retrieval with sodium citrate buffer heated in a microwave to 95 C for 3 min, then incubated for 30 min with blocking buffer containing 2% goat serum, 1% bovine serum albumin, 0.1% triton-X 100, 0.05% tween-20, and 0.05% sodium azide. Rabbit polyclonal antisera to *P. gingivalis* W83, which was previously validated for its specificity to the microbe [24], was used at a dilution of 1:2000. Pre-immune serum from the same rabbit was used as an isotype control. Tissues were incubated with the antibody preparations overnight at 4 C. For detection, ALEXA 647 labeled goat anti-rabbit antibody (catalog # A32733, ThermoFisher Scientific, Waltham, MA, USA) was used at a concentration of 1 μg/ml diluted in blocking buffer. Tissue sections were covered with glass coverslips mounted with Prolong Gold Antifade Reagent with DAPI (catalog # P36931, ThermoFisher Scientific, Waltham, MA, USA).

### Cell culture

Dermal microvascular endothelial cells of neonatal origin (HD-MVEC) were obtained from Lifeline Cell Technology (catalog # FC-0042, Frederick, MD, USA). For all experiments, HD-MVEC cultures were maintained in VascuLife® EnGS-MV medium (Lifeline Cell Technology, Frederick, MD, USA) and were used within 9 passages. Unless stated otherwise, cells were seeded at a density of 1 × 10^5^ cells per 12-well cell culture plate or glass coverslips 24 h before inoculation with *P. gingivalis* and maintained at 37°C/5% CO_2_.

### Endothelial branching assays

Endothelial branching assays were performed as previously described [12, 40] with the following modifications. HD-MVEC that were cultured in T25 flasks were inoculated with sterile medium (control) or *P. gingivalis* at an MOI of 100. At 3 h post-inoculation, HD-MVEC were washed and treated with Accutase™ cell detachment solution (Innovative Cell Technologies, San Diego, CA) and transferred to thinly coated Matrigel culture plates. Uninfected control and *P. gingivalis* inoculated HD-MVEC were then seeded at a density of 2 × 10^4^ cells per cm^2^ and maintained in EnGS-MV medium at 37°C/5% CO2 until termination of experiment. After 24 h, cells were evaluated for endothelial network formation.

HD-MVEC viability was evaluated by LIVE/DEAD® Viability/Cytotoxicity Kit (catalog # L3224, ThermoFisher Scientific, Waltham, MA, USA). Cells were seeded onto sterile coverslips 24 h before inoculation with *P. gingivalis* at an MOI of 100. After 1.5 h, cells were gently washed twice with sterile media, then maintained in EnGS-MV media containing 300 ug/ml gentamicin and 400 ug/ml metronidazole. HD-MVEC were harvested at 24 h post-infection, and processed according to the manufacturer’s instructions. For viability assessment, at least 3 random images from each biological replicate were taken at 20x magnification with an Olympus Deconvolution microscope (Olympus Corp.). number of dead and live cells were recorded from each image, and the sum of dead cells within each replicate was divided by the total number of cells (dead + live) and expressed as a percent.

HD-MVEC were evaluated for apoptosis by activation of Caspase 3 and 7, which was detected with CellEvent™ Caspase-3/7 Green Detection Reagent (catalog # C10723, ThermoFisher Scientific, Waltham, MA, USA). Cells were seeded onto glass coverslips and inoculated as described above. In addition, a positive control was included that consisted of HD-MVEC being treated with 1 mM H_2_O_2_ for 24 h.

### W83 invasion and persistence in HD-MVEC

All invasion experiments of HD-MVEC were performed as previously described [18,22]. Briefly, HD-MVEC were seeded at a cell density of 1 × 10^5^ cells per well in 12-well cell culture plates, 18 h later they were washed 3 times with antibiotic-free EnGS-MV medium before inoculation with bacteria. Initial invasion assays were performed by two methods as previously described [25]. Briefly, HD-MVEC were inoculated with *P. gingivalis* at an MOI of 100 (no spin protocol). In the second method, HD-MVEC were chilled on ice for 10 min before inoculation with *P. gingivalis* at an MOI of 100 and centrifuged at 1000 x g for 10 minutes at 4°C. Inoculated cells were then transferred to 37°C/5% CO2 incubator, which was considered time zero. This approach allowed for synchronization of bacteria with host cells [22]. At 1.5 h post-inoculation, cell cultures were washed 3 times with EnGS-MV medium and fresh media supplemented with 300 µg/ml gentamicin and 400 µg/ml metronidazole (Sigma-Aldrich) was added to kill any remaining extracellular bacteria. Microbial killing of extracellular bacteria was confirmed by culture as previously described [22]. The cells were maintained in this medium until termination of the experiment. For assessment of intracellular persistence, antibiotic treatment was maintained until cell harvest. For assessment of microbial ability to exit host cells, cells were pulse treated with antibiotics for 1 h so that at 2.5 h post-inoculation, HD-MVEC cultures were washed and were maintained in antibiotic-free EnGS-MV medium thereafter. At the time of cell harvest, HD-MVEC were lysed by incubation in sterile-distilled water for 20 min at 37°C/5% CO2. Cell lysates were serially diluted in sterile PBS, and the number of viable *P. gingivalis* organisms was enumerated by culture.

Antibody-based blockade of ICAM-1 [26] was used to determine if *P. gingivalis* utilized this receptor for gaining entry into HD-MVEC. Briefly, HD-MVEC were pre-incubated with 20 μg/ml of a monoclonal mouse anti-ICAM-1 (catalog # MA5407, clone IA29, ThermoFisher Scientific, Waltham, MA, USA) or a mouse IgG isotype control (catalog # 31,903, ThermoFisher Scientific, Waltham, MA, USA) for one hour before at 37° C and 5% CO_2_. Antibody clone 1A29 recognizes the external domain of ICAM-1 and was previously shown to functionally block ligand binding to this receptor [27,28]. At the end of the hour, HD-MVEC were inoculated with 100 MOI of *P. gingivalis* W83 using the spin inoculation protocol described above. At 2.5 h post-inoculation, HD-MVEC were lysed and the number of internalized *P. gingivalis* were enumerated by culture.

### P. *gingivalis*/HD-MVEC vesicle colocalization studies

Colocalization of *P. gingivalis* with autophagosomes, early endosomes, or late endosomes was achieved by transduction with fluorescently tagged vectors inoculated at an MOI of 10 forty-eight hours before infection. Autophagosomes were tagged with green fluorescent protein (GFP)-light chain three (LC3) packaged in an adenovirus expression system (Welgen, Inc, Worcester, MA, USA). Both Rab5 (early endosome) and Rab7 (late endosome) were tagged with red fluorescent protein (RFP) packaged in a baculovirus system (CellLight® BacMam 2.0, Life Technologies, Grand Island, NY). Caveolae were labeled with a rabbit polyclonal antibody to caveolin-1 used at a dilution of 1:200 (catalog # ab18199, Abcam, Cambridge, MA). Late phagosomes/lysosomes were labeled with rabbit polyclonal antibody to LAMP1 used at a 1:200 dilution (catalog # SC-5570, Santa Cruz Biotechnology, Inc, Dallas, TX). Goat anti-mouse or anti-rabbit antibodies labeled with ALEXA 594 (absorption 590, emission 617) or ALEXA 647 (absorption 650, emission 665) (Life Technologies, Grand Island, NY) were used as secondary antibodies.

HD-MVEC were inoculated with *P. gingivalis*, and incubated for 2.5, 6, and 24 h as described above. At the end of each time point, cells were fixed with 4% paraformaldehyde dissolved in phosphate buffered saline (PBS). After washing with PBS, fixed cells were first blocked for 30 minutes with blocking buffer (described above) prior to the addition of antibodies. All cells were washed three times with PBS before mounting with ProLong® Gold Antifade reagent with DAPI (Invitrogen™). HD-MVEC were visualized with an Olympus DSU-IX81 Spinning Disc Confocal microscope and images were captured with Slidebook software (Olympus, Center Valley, PA). At least three images at 20X magnification were obtained from each specimen. Final processing of images was performed with ImageJ software (US National Institutes of Health, Bethesda, MD).

Colocalization analysis of *P. gingivalis* with each specific marker was performed as previously described [22]. Briefly, the total number of internalized bacteria within intact cells of each image were counted (i.e. bacteria colocalized with the marker of interest + internalized bacteria not associated with any marker). All values from each image obtained from the same biological replicate were added and the proportion of *P. gingivalis* associated with each marker was reported as the percent of total internalized bacteria.

### Data analysis

Data was analyzed by unpaired Student’s t-test or one way ANOVA followed by Tukey’s multiple comparisons test. Analyses were performed with Prism 8.12 Software (GraphPad). For all analysis, P < 0.05 was considered significantly different.

## Results

### P. *gingivalis-*induced periodontitis includes vasculopathy and microbial invasion of gingival tissue

**Figure 1.**
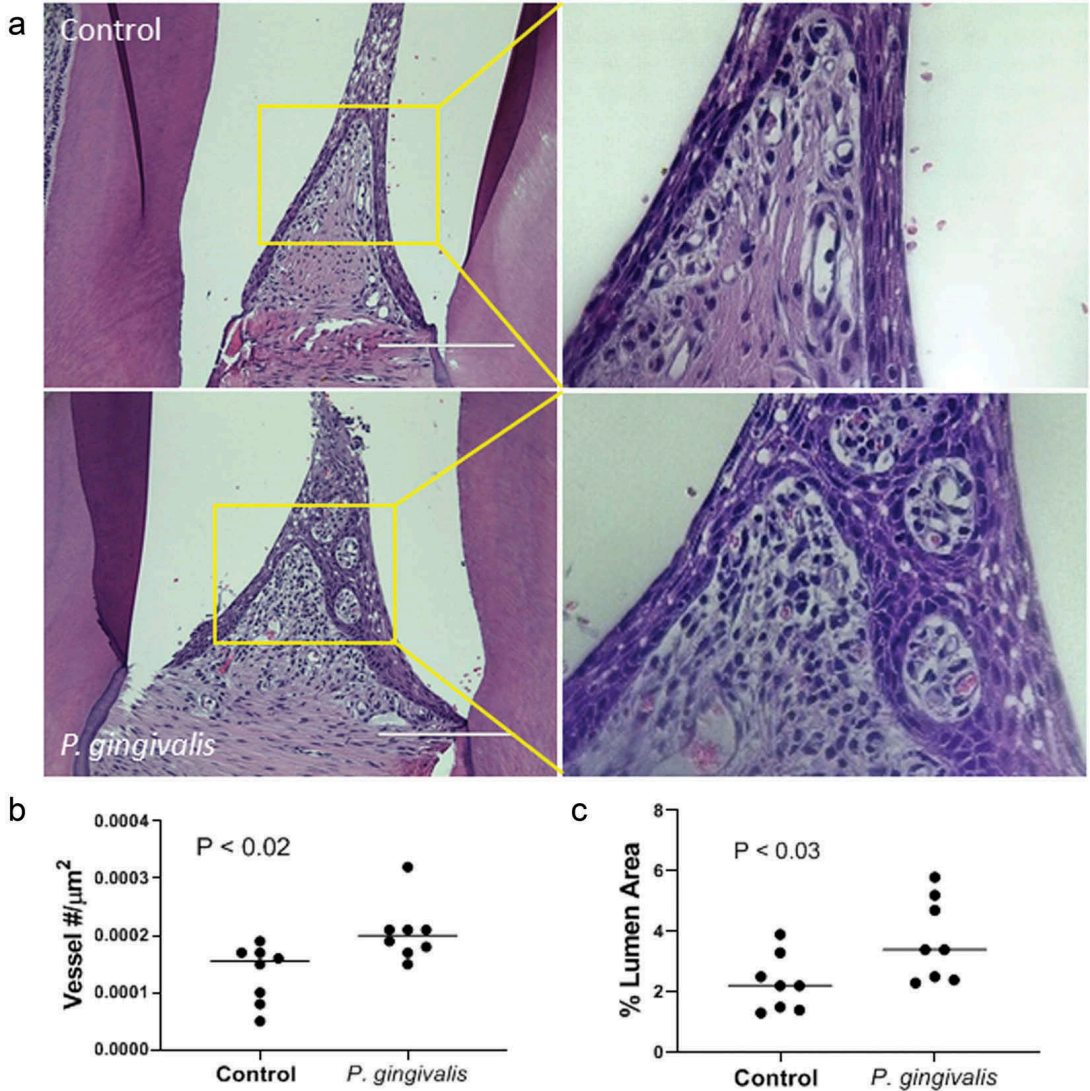
*P. gingivalis*-induced changes in rat interdental papillae. (a) Representative H & E stained tissue sections from sham control and *P. gingivalis* inoculated animals. Panels to the right of each image are magnified images of the boxed region. Scale bars = 200 μm. (b) Subgingival microvascular density expressed as vessel number/tissue area (μm^2^), and (c) microvascular dilatation (total vessel lumen area/tissue area)

The interdental papillae of infected animals displayed lesions consistent with chronic inflammation [2,29], which included increased leukocyte infiltrates subadjacent to the subgingival epithelium and a prominent microvasculature (Figure 1(a), magnified inset). We utilized morphometry to quantify changes in the subgingival vascular structure. Density (Figure 1(b)) was measured as the number of capillary loops/um^2^ of the interdental papilla, which was greater in *P. gingivalis* infected animals than controls: 2.1 × 10^−4^ ± 5 x 10^−5^ vs. 1.33 × 10^−4^ ± 5 x 10^−5^, respectively, expressed as mean ± SD (Figure 1(b)). The lumen diameter of the subgingival microvasculature was also greater in *P. gingivalis* infected animals compared to controls: mean % ± SD was 3.7 ± 1.4% for *P. gingivalis* infected vs. 2.3 ± 0.9% for untreated (Figure 1(c)).

**Figure 2.**
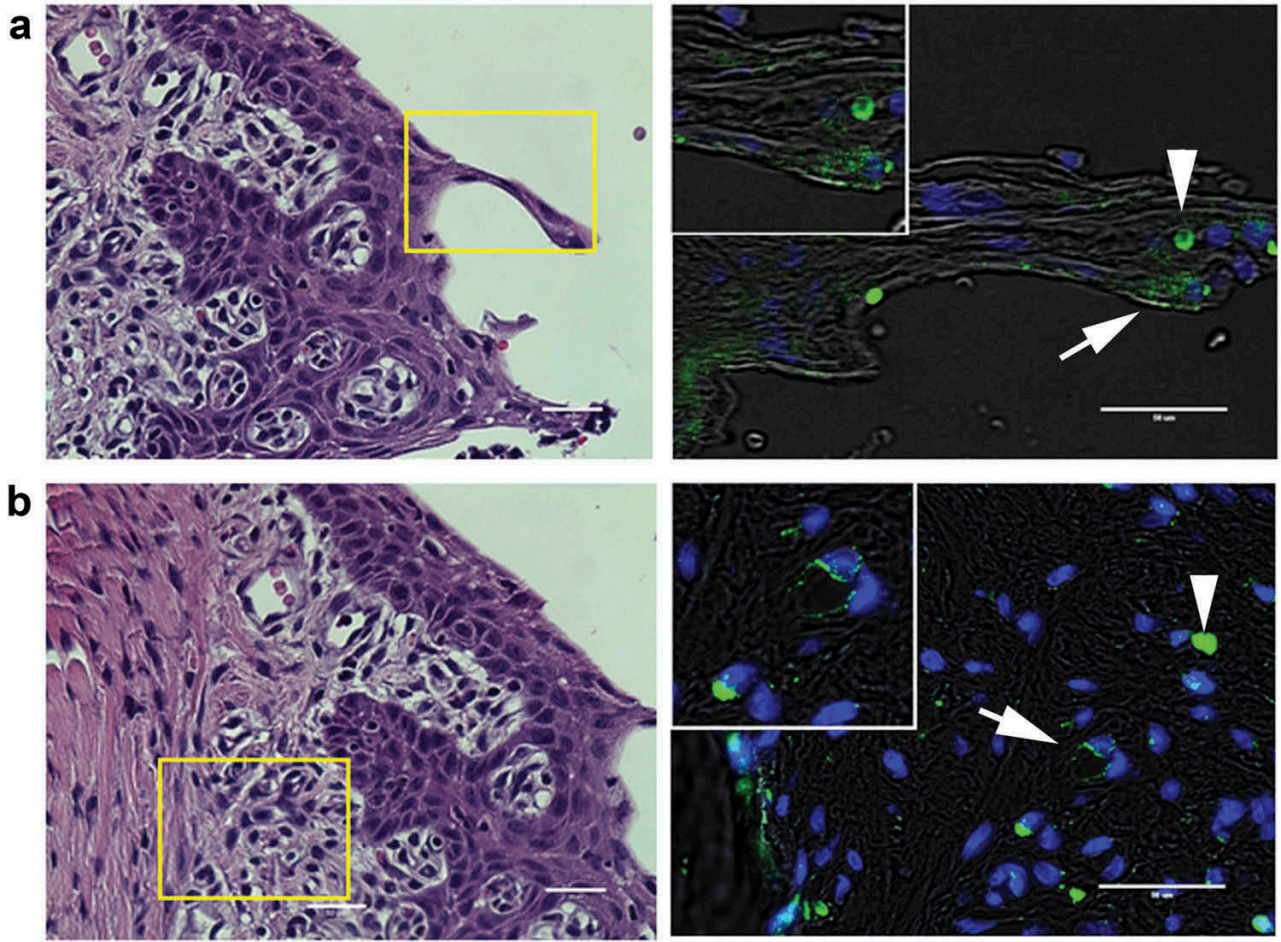
Representative images of *P. gingivalis* staining in the gingival tissue of interdental papillae. (a) H & E stained section of the gingival epithelium, showing the approximate region (boxed area) where *P. gingivalis* was detected by immunostaining (right panel). White arrow points to a *P. gingivalis* positive area (pseudocolored green) in the gingival epithelial layer that is also shown in the magnified inset. White arrowhead points to an autofluorescent red blood cell present in the section. (b) H & E stained section of the subgingival connective tissue matrix, boxed region indicates approximate location of where *P. gingivalis* positive subgingival capillaries were detected (white arrow, pseudocolored green) that is also included in the top magnified inset. The white arrowhead points to an autofluorescent red blood cell present in the section. Transillumination was used to demarcate tissue architecture and nuclei were stained with DAPI (blue). Scale bars are 50 μm

In infected animals, *P. gingivalis* was detected in the epithelial layer (Figure 2(a)) as well as the subepithelial tissue matrix in areas of increased inflammatory cell infiltration (Figure 2(b)). A less common but consistent observation was detection of *P. gingivalis* in association with subepithelial capillaries (Figure 2(b)). *P. gingivalis* staining was not observed in isotype controls nor in uninfected control specimens (Supplement Figure S2), confirming the specificity of the antibody.

### P. gingivalis invades and persists in HD-MVEC

**Figure 3.**
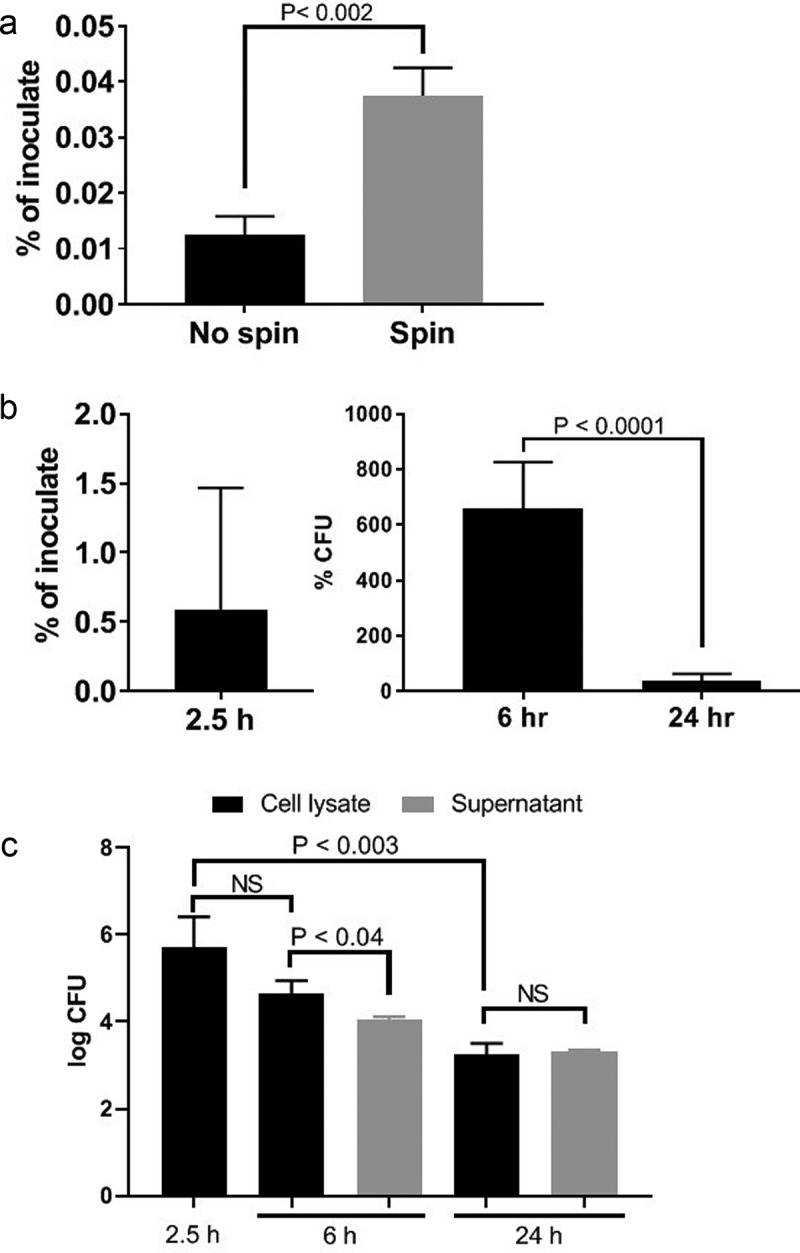
Invasion by no spin and spin inoculation (a), invasion and intracellular persistence of *P. gingivalis* within HD-MVEC maintained under constant antibiotic (b) or pulse antibiotic (c) treatment. (a) The proportion of bacterial inoculates enumerated from HD-MVEC cell lysates collected at 2.5 h post-inoculation (n = 4). (b) *P. gingivalis* inoculation was performed by centrifugation and antibiotics were added to HD-MVEC cultures at 1.5 h post-inoculation and maintained until cell harvest. Percent at 2.5 h post-inoculation represents the mean proportion of inoculate that was enumerated from HD-MVEC cell lysates at 2.5 h post-inoculation. Values at 6 and 24 h post-inoculation were determined by dividing the CFU obtained at each time point by the average CFU obtained at 2.5 h post-inoculation (i.e. invaded cells). Values represent the mean ± SD (n = 6) from two independent experiments. (c) *P. gingivalis* inoculation was performed by centrifugation. At 1.5 h PI, HD-MVEC were treated with antibiotics for 1 h. Values are expressed as the mean log CFU ± SD (n = 3)

We next determined if *P. gingivalis* was able to invade and persist in HD-MVEC since it does so in human coronary arterial endothelial cells (HCAEC) and human umbilical endothelial cells (HUVECS) and [18,20,30]. Invasion assays were performed under constant antibiotic pressure so that cultures only retrieved internalized bacteria. No live bacteria were isolated from cell culture supernatants collected at 2.5 h post-inoculation (data not shown). Consistent with previous invasion studies with endothelial cells [25], live bacteria were isolated from HD-MVEC cell lysates with yields being significantly greater from cells that underwent the spin inoculation protocol (Figure 3(a)). Remaining invasion and persistence experiments were performed using the spin inoculation protocol. At 2.5 h post-inoculation, the mean ± SD log CFU (% of corresponding inoculate) retrieved from HD-MVEC lysates was 4.23 ± 0.78 (0.58 ± 0.9%) (n = 6, combined from 2 independent experiments). The mean log CFU ± SD cultured from cell lysates obtained at 6 and 24 h post-inoculation were 4.39 ± 4.1 and 3.14 ± 3.2, respectively (n = 6, from 2 independent experiments). The number of internalized bacteria at 6 and 24 h post-inoculation were normalized by dividing the CFU obtained from these time points by the corresponding mean CFU obtained at 2.5 h post-inoculation, which we considered as time zero (Figure 3(b)). We found 6-fold more internalized culturable bacteria at 6 h post-inoculation than at 2.5 h suggesting that *P. gingivalis* was replicating within these cells. However, by 24 h post-inoculation, the proportion of live *P. gingivalis* within HD-MVEC was reduced.

In order to establish if live *P. gingivalis* were released from HD-MVEC, we repeated the invasion assay under antibiotic pulse (1 h) treatment (Figure 3(c)). Cells were inoculated with *P. gingivalis* by the spin inoculation method. The log CFU from HD-MVEC cell lysates collected at 2.5 h post-inoculation was 5.7 ± 0.7 (mean ± SD). Similar to human coronary arterial endothelial cells [18], viable *P. gingivalis* was retrieved from cell culture supernatants at both 6 and 24 h post-inoculation. However, the CFU of live *P. gingivalis* retrieved from 6 h post-inoculation cell culture supernatants was less than what was isolated from cell lysates, approximately 22% of what was retrieved from cell lysates (Figure 3(c)), suggesting that extracellular bacteria do not survive as well or do not replicate in the supernatant as they do within the cell. By 24 h post-inoculation, the CFU of *P. gingivalis* isolated from cell culture supernatants and cell lysates were equivalent (Figure 3(c)).

### In HD-MVEC, *P. gingivalis* primarily traffics through ICAM-1 positive vesicles

**Figure 4.**
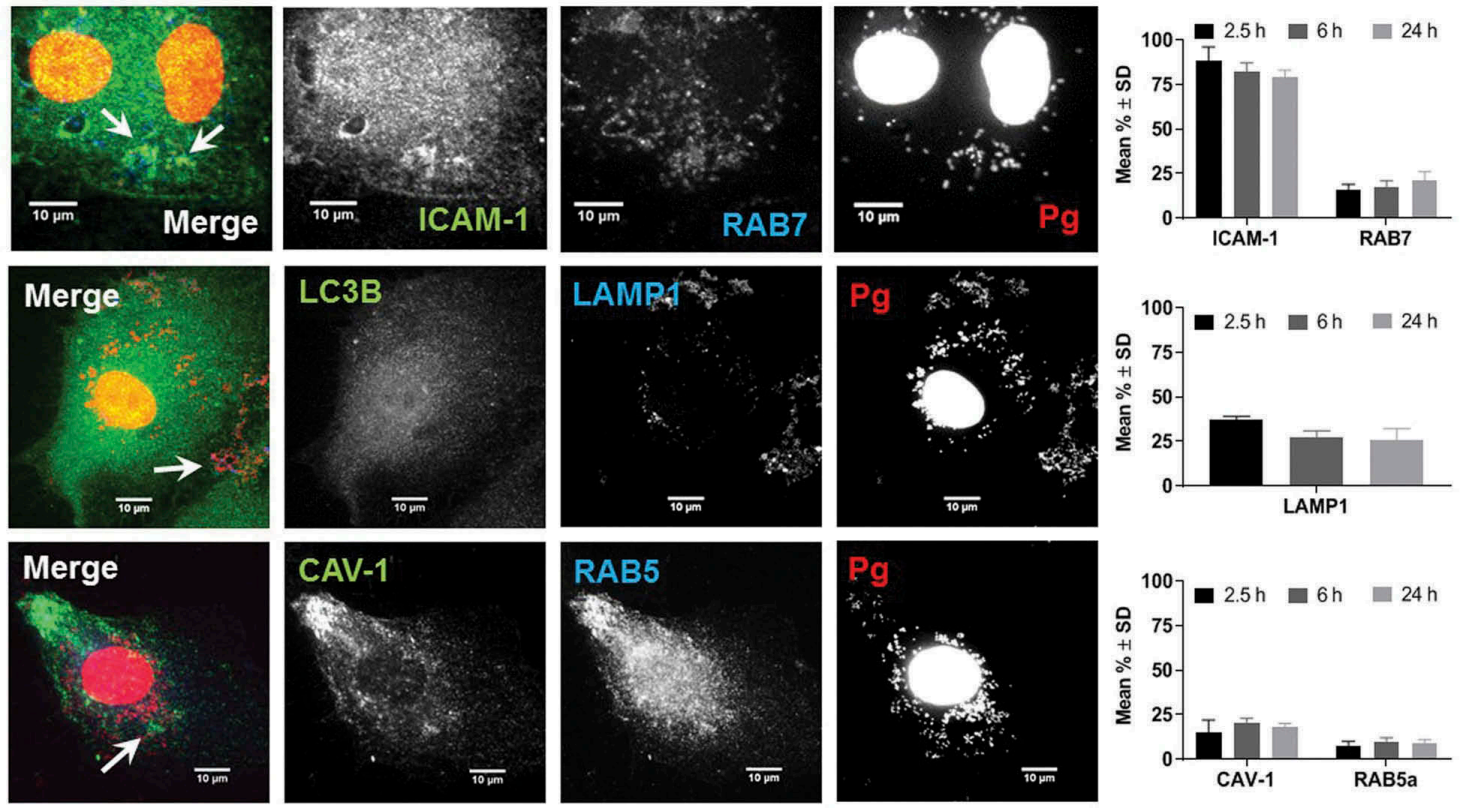
Colocalization of *P. gingivalis* (DAPI) with ICAM-1, Rab7, LAMP1, Caveolin-1 (CAV), and Rab5. Representative images are from 6-h PI time points. Merged images contain all three channels displayed within the same row. DAPI stained *P. gingivalis* is pseudocolored red, and Rab7, LAMP1, and Rab5 are pseudocolored blue to improve visualization of bacterial colcocalization with each respective marker (white arrows). Graphs display the number of internalized *P. gingivalis* expressed as mean % ± SD from 3 independent experiments

Depending on host cell type, microbial strain, and MOI, *P. gingivalis* invades and traffics through a variety of cell compartments [18,26,31]. For example, *P. gingivalis* gains entry into human oral epithelial cells by engagement of ICAM-1 and caveolae that will transit to endosomes [26]. *P. gingivalis* enters human coronary arterial endothelial cells (HCAEC) via endosomes [18,31]. At an MOI of 100, *P. gingivalis* strains W83 and 381 traffic through the autophagic pathway in HCAEC, but strains A7436 or 33277 do not [18]. When HCAEC are supersaturated with an MOI of 10^4^, the majority of *P. gingivalis* (W83, 381, and 33277) are found in the lysosome compartment rather than the autophagosome [31]. Using an MOI of 100, we therefore assessed colocalization of *P. gingivalis* with ICAM-1, caveolin-1, early endosomes (RFP-Rab5), late endosomes (RFP-Rab7), lysosomes (LAMP-1) and autophagosomes (GFP-LC3B) over 2.5, 6, and 24 h post-inoculation (Figure 4). Experiments were performed under constant antibiotic pressure as already described [22].

Regardless of the duration of infection, the majority of internalized *P. gingivalis* (79–88%) was found in ICAM-1 positive vesicles. Moreover, ICAM-1 clustering that was observed in W83 infected cells was not present in uninfected control HD-MVEC (Figure S3 in Supplement file). Only 10–39% of internalized bacteria were found in caveolae-derived vesicles, early (Rab5) and late endosomes (Rab7), and/or lysosomes (LAMP-1) (Figure 4). Regardless of the duration of infection, *P. gingivalis* was never found in autophagosomes (Supplement file, Figure S4). This could not be attributed to failure of vector expression since GFP-LC3B protein and puncta presumed to be autophagosomes were seen in transduced HD-MVEC (Figure 4, and Figure S3 in supplement file). Thus, our data suggest that *P. gingivalis* does not traffic through the autophagic pathway in HD-MVEC.

The mean total number of internalized bacteria counted in the HD-MVEC microscopic images used for *P. gingivalis* trafficking experiments was 75 ± 40 (mean ± SD per 3 cells in each field) at 2.5 h post-inoculation, which was significantly less than the number counted at the 6 h (197 ± 72 per cell) and 24 h (190 ± 113 per cell) time points (P < 0.05). The increase in bacterial numbers from the 2.5 h to 6 h time points was similar to the results obtained from the invasion and persistence assays (Figure 3(b)). However, we saw no difference between bacterial counts from the 6 and 24 h time points, which deviated from culture results shown in Figure 3(b).

**Figure 5.**
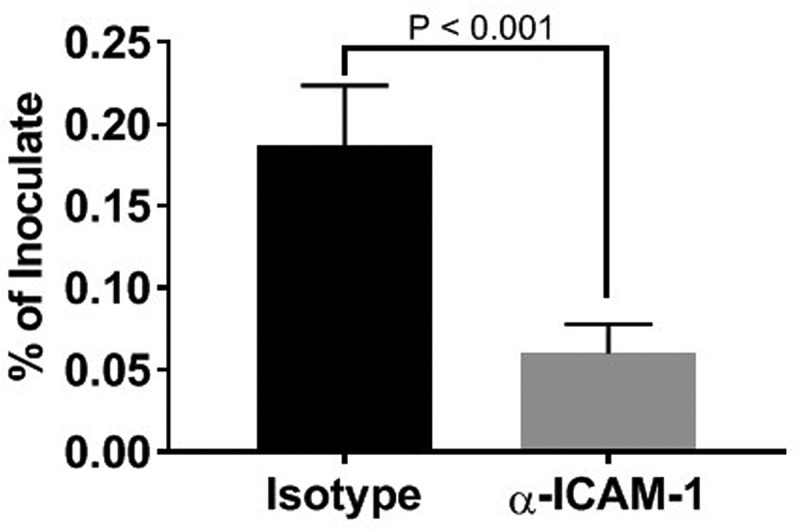
Inhibition of *P. gingivalis* invasion into HD-MVEC by anti-ICAM-1 antibody. Values represent the mean ± SD (n = 6) from two separate experiments. Percent values were determined by dividing the CFU of internalized bacteria by the CFU of the corresponding inoculate

ICAM-1 clustering can serve as a point of entry into endothelial cells [32], and *P. gingivalis* has been shown to enter KB cells through engagement of this receptor [26]. Since the majority of *P. gingivalis* colocalized with ICAM-1 positive vesicles, we evaluated the effect of an ICAM-1 blockade on *P. gingivalis* invasion of HD-MVEC using a commercial antibody validated for this use (Figure 5). At 2.5 h post-inoculation, the mean percent ± SD of inoculate retrieved from lysed cells pretreated with the isotype control was 0.2 ± 0.04%, which was not significantly different from untreated cells (0.58 ± 0.9%). Pretreatment of HD-MVEC with mouse monoclonal antibody to ICAM-1 significantly reduced the number of intracellular *P. gingivalis* (P < 0.0001); 0.06 ± 0.02% of inoculate in pre-anti-ICAM-1 treated cells vs. 0.2 ± 0.04% in pre-isotype control treated cells.

**Figure 6.**
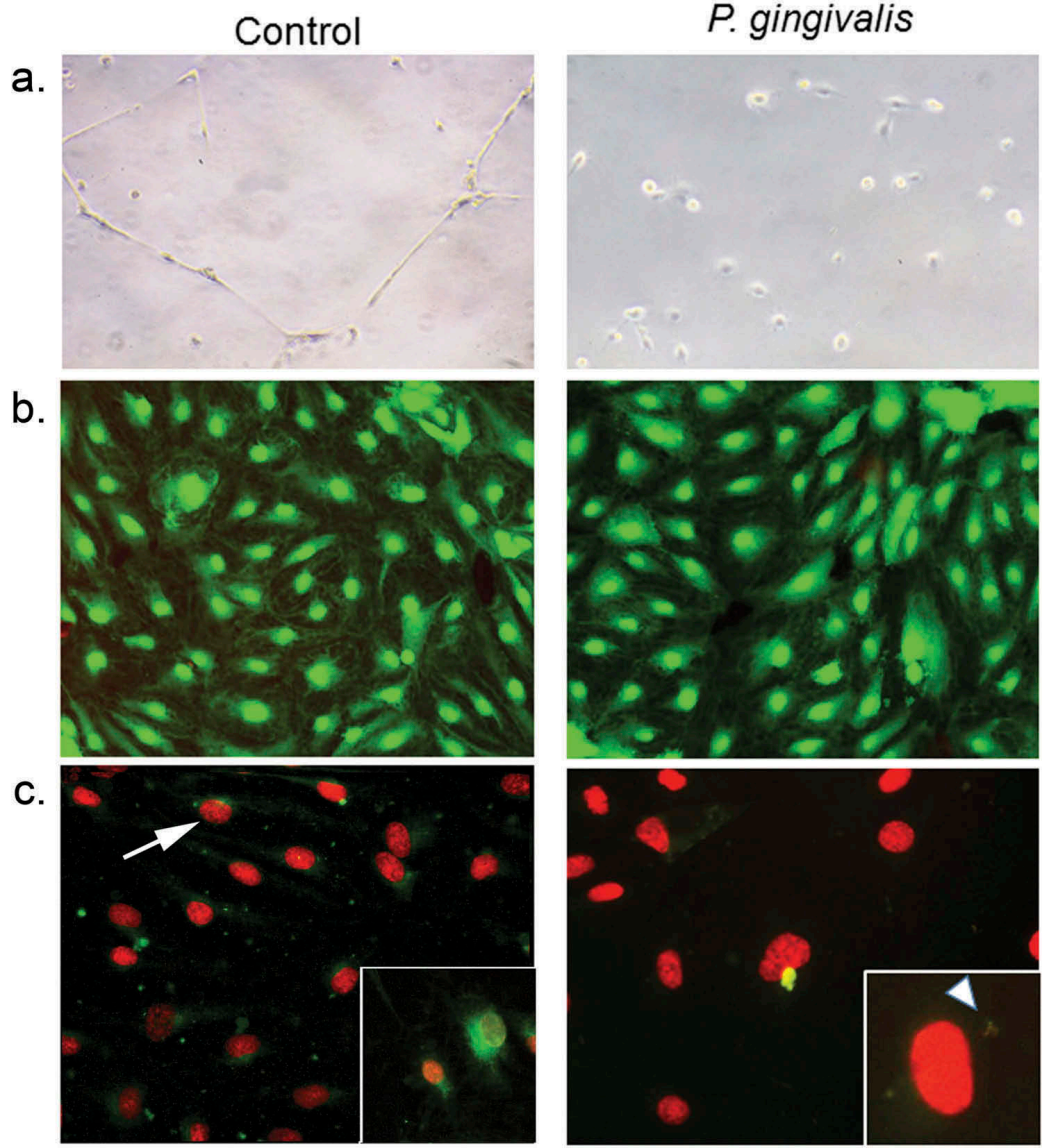
Impact of *P. gingivalis* infection on HD-MVEC network formation (a), viability (b), and apoptosis(c). (a) HD-MVEC network formation on Matrigel after 24 h. (b) HD-MVEC viability (green) assessed with LIVE/DEAD® Viability/Cytotoxicity Kit, dead cells are labelled red. (c) Apoptosis detected by activation of caspase 3/7 (green, white arrow) as shown in the magnified inset in control panel, which is an H_2_O_2_ positive control (arrow). Magnified inset in (c) *P. gingivalis* panel shows internalized bacteria (white arrowhead) confirming that cells were infected

### Infection with *P. gingivalis*disrupts *HD-MVEC* tubular formation

We utilized an endothelial branching assay [12] to evaluate the impact of *P.gingivalis* infection on the angiogenic capacity of HD-MVEC [33]. Cells grown in flasks were first inoculated with sterile media or infected with *P. gingivalis* for 3 h before assessing tube formation, which was done by transferring cells to Matrigel and monitoring for 24 h. Consistent with previous work [12], uninfected HD-MVEC cells readily formed branching networks by 24 h after seeding on Matrigel. In contrast, HD-MVEC infected with *P. gingivalis* were unable to form branching networks (Figure 6(a)). We then assessed cell viability since gingipain extracts from *P. gingivalis* have been shown to induce HD-MVEC apoptosis [34], which could be a potential cause of impaired tube formation. We found no difference in the proportion of dead HD-MVEC between control and *P. gingivalis* infected groups (n = 4 combined from two separate experiments: 1.14% ± 0.66 in control vs. 1.04% ± 0.06 in *P. gingivalis* positive cells, Figure 6(b)). Similarly, *P. gingivalis* infected HD-MVEC did not show an increase in caspase 3/7 activation at 24 h post-inoculation (Figure 6 C), indicating that the lack of tube formation could not be attributed to cell death.

## Discussion

Both transcellular and paracellular invasion of gingival epithelium by periodontopathogenic bacteria are recognized as important mechanisms of severe and persistent periodontitis [1,9,35,36]. It is less clear what role periodontitis-associated changes in the subgingival microvascular network have on the disease process. It has been proposed that alterations in the subgingival vasculature such as upregulation of cell adhesion molecules may be a compensatory mechanism that increases the supply of both plasma defense factors and leukocyte extravasation to the tissues, which should assist in elimination of infection [2]. On the other hand, these changes may also impede recovery from disease by promoting tissue biofilms and microbial persistence in the gingival and subgingival tissues [1]. For instance, ICAM-1 mediated endocytosis in human umbilical vein endothelial cells has been shown to be exploited by the Gram-negative bacterium, *Bartonella hensilae* as a means to gain entry into host cells and support its persistence [37]. In this study, we provide both *in vivo* and *in vitro* evidence that *P. gingivalis* W83 disrupts the subgingival microvasculature, producing pathologic changes consistent with human periodontal disease (i.e. increased vascular density and vessel dilatation). Moreover, we show that *P. gingivalis* W83 utilizes ICAM-1 mediated entry into HD-MVEC, thereby allowing it to persist and disseminate within the gingival tissue.

The earliest events of microbial invasion of the gingiva are through the epithelial layer via transcellular and/or paracellular trafficking [7]. In rats, we found the *in situ* distribution of *P. gingivalis* W83 was similar to that in human gingival specimens from chronic periodontitis patients [10]. That is, *P. gingivalis* was most often detected in the gingival epithelium and gingival tissue matrix. The organism was also detected in association with subgingival capillaries which, although less frequent, was accompanied by colonization of the tissue matrix with an increased leukocyte infiltrate. This distribution suggests that microbial invasion of the microvasculature may be assisted by the vasculature responding to local inflammation.

*In vivo* infection with *P. gingivalis* produced lesions consistent with human periodontal disease including dilated tortuous subgingival microvasculature, consistent with chronic periodontal disease [2]. Since our animals were also colonized with oral commensal bacteria we cannot disregard the possibility that the changes we observed with *P. gingivalis* infection is partially a consequence of oral dysbiosis and overgrowth of oral commensal bacteria [38,39]. Nevertheless, *P. gingivalis* singly disrupted the ability of HD-MVEC’s to form a vascular network in Matrigel. It is curious that this was not due to endothelial cell death. The disruption of angiogenesis by W83 without killing host cells may be a mechanism for the infection and/or a means of entering the systemic circulation of the host.

Endothelial cells display tissue specific morphologic and functional heterogeneity [41]. Since our focus was on *P. gingivalis* interactions with microvascular endothelium, we chose HD-MVEC for our *in vitro* model system due to their similarity to gingival microvascular endothelial cells [12]. We found that HD-MVEC responded differently to *P. gingivalis* infection compared to endothelial cells derived from large vessels. For instance, *P. gingivalis* infection of HD-MVEC did not induce apoptosis or cell death as has been observed in HUVECS [21]. Moreover, we found that internalization and trafficking of *P. gingivalis* was markedly different in HD-MVEC compared to human coronary artery endothelial cells (HCAEC) [18,22,42]. For example, *P. gingivalis* strain W83, which was used in this study, predominantly traffics through the autophagic pathway during invasion of HCAEC [18,22]. Although there was evidence that transduced HD-MVEC expressed GFP-LC3B protein, internalized *P. gingivalis* was never found in LC3B positive vesicles. This suggests that in HD-MVEC, *P. gingivalis* does not traffic through this pathway. Instead, we found that infection with *P. gingivalis* induced clustering of ICAM-1 in HD-MVEC and that the majority of internalized bacteria co-localized with ICAM-1 positive vesicles. Moreover, antibody-based blockade of ICAM-1 significantly reduced the entry of W83 into HD-MVEC. Taken together, ICAM-1 may be important for microbial entry into HD-MVEC. In epithelial cells, it has been shown that *P. gingivalis* strain 33277 gains entry into these cells via fimbrial engagement of ICAM-1 and caveolin-1 [26].

A distinctive feature of ICAM-1 mediated endocytosis in HUVECs and microvascular endothelial cells, is that it can occur independently of caveolin and clathrin [32,43,44]. This unique pathway is triggered by surface clustering of ICAM-1 that initiates its internalization [32]. Following internalization, aggregated ICAM-1 positive endosomes recycle back to the cell surface (i.e. luminal side), transport across the cell to the basolateral side (transcellular), or merge with a lysosome where contents can be degraded [32,44,45]. Our study indicates that *P. gingivalis* may be primarily trafficking through ICAM-1-mediated endocytosis. W83 upregulates ICAM-1 expression in endothelial cells [46], which increases the opportunity for clustering of this molecule and initiating endocytosis independent of caveolin and clathrin. Indeed, we found aggregates of ICAM-1 in infected HD-MVEC that were not present in uninfected cells. Furthermore, the majority of internalized W83 was in ICAM-1 positive endosomes, but less than 25% colocalized with caveolin-1 at any of the examined time points. Moreover, we retrieved live W83 from HD-MVEC supernatants that were pulse treated with antibiotics suggesting that bacteria may be exiting out of HD-MVECS at both 6 and 24 h post-inoculation.

Some internalized *P. gingivalis* was also directed to lysosomes since we also found bacteria co-localizing with Rab7 and LAMP1. The significant decrease in the number of live bacteria cultured from 24 hour cell lysates would indicate that some bacteria were being degraded. However, it is also possible that internalized *P. gingivalis* converted to a viable but not cultivable (VBNC) state, which would allow it to persist. The similar numbers of internalized *P. gingivalis* detected at 6 and 24 h post-inoculation in microscopic images would lend support to this view. It is also possible that W83 can prevent the final lysosome fusion event, thereby establishing a lysosomal environment that is defective in degradation [47]. Additional studies will be required to determine the ultimate fate of W83 in HD-MVECs.

The microvasculature subadjacent to oral epithelium expresses ICAM-1 in apparently healthy gingiva [48], and its expression increases during periodontitis [49]. Whether or not endothelial expression of ICAM-1 helps to protect the tissues from invasion by periodontal bacteria is unclear. In this study, we show that microbial clustering of ICAM-1 on HD-MVEC may inadvertently provide a means for periodontopathogenic bacteria to enter the microvascular endothelium and persist within the subgingival tissue.

In conclusion, experimental infection with *P. gingivalis* W83 in a rat model induced changes in the subgingival microvasculature that are characteristic of human periodontal disease [2,3]. Our *in vitro* experiments demonstrated that W83 disrupts angiogenesis, illustrating that invasion of the gingival endothelium by *P. gingivalis* likely results in microvascular dysfunction. In addition, unlike entry and trafficking in endothelial cells from large vessels, W83 enters microvascular endothelial cells via ICAM-1 where it can be released from endothelial cells and re-infect neighboring cells [25] or reside within tissue biofilms. Alternatively, *P. gingivalis* can enter the lysosomal pathway, where it is degraded or enters the VBNC state. All scenarios could be significant in the progression of periodontal disease and the establishment of *P. gingivalis* in tissues throughout the body.

## Supplementary Material

Supplemental Material

## Data sharing

The data that support the findings of this study are available from the corresponding author, [LR], upon reasonable request.
