# Supplementary material for: Porphyromonas gingivalis W83 traffics via ICAM1 in microvascular endothelial cells and alters capillary organization in vivo
Source: J Oral Microbiol. 2020 Mar 26;12(1):1742528. doi: 10.1080/20002297.2020.1742528 (PMC7170297; doi:10.1080/20002297.2020.1742528)
Supplement: Supplemental Material [file ZJOM_A_1742528_SM4810.docx]

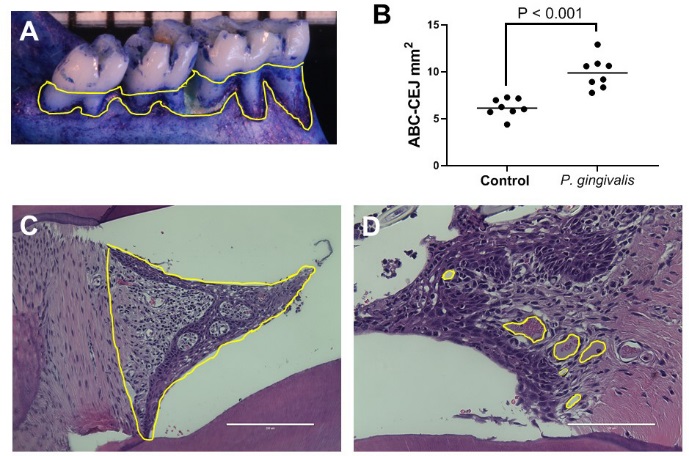


**Figure S1. Morphometric analysis of periodontal disease in SD rats.** A) Representative image of a methylene blue stained, defleshed jaw from a *P. gingivalis* infected rat. The yellow line demarcates the border of the cemento-enamel junction to alveolar bone crest that was used for measuring alveolar bone loss in panel B. C) Demonstrates how the region of interest (yellow line) within the interdental papilla was determined for morphometric analysis. D) Shows how lumen area measurements (yellow line tracings) within the interdental papilla were taken.


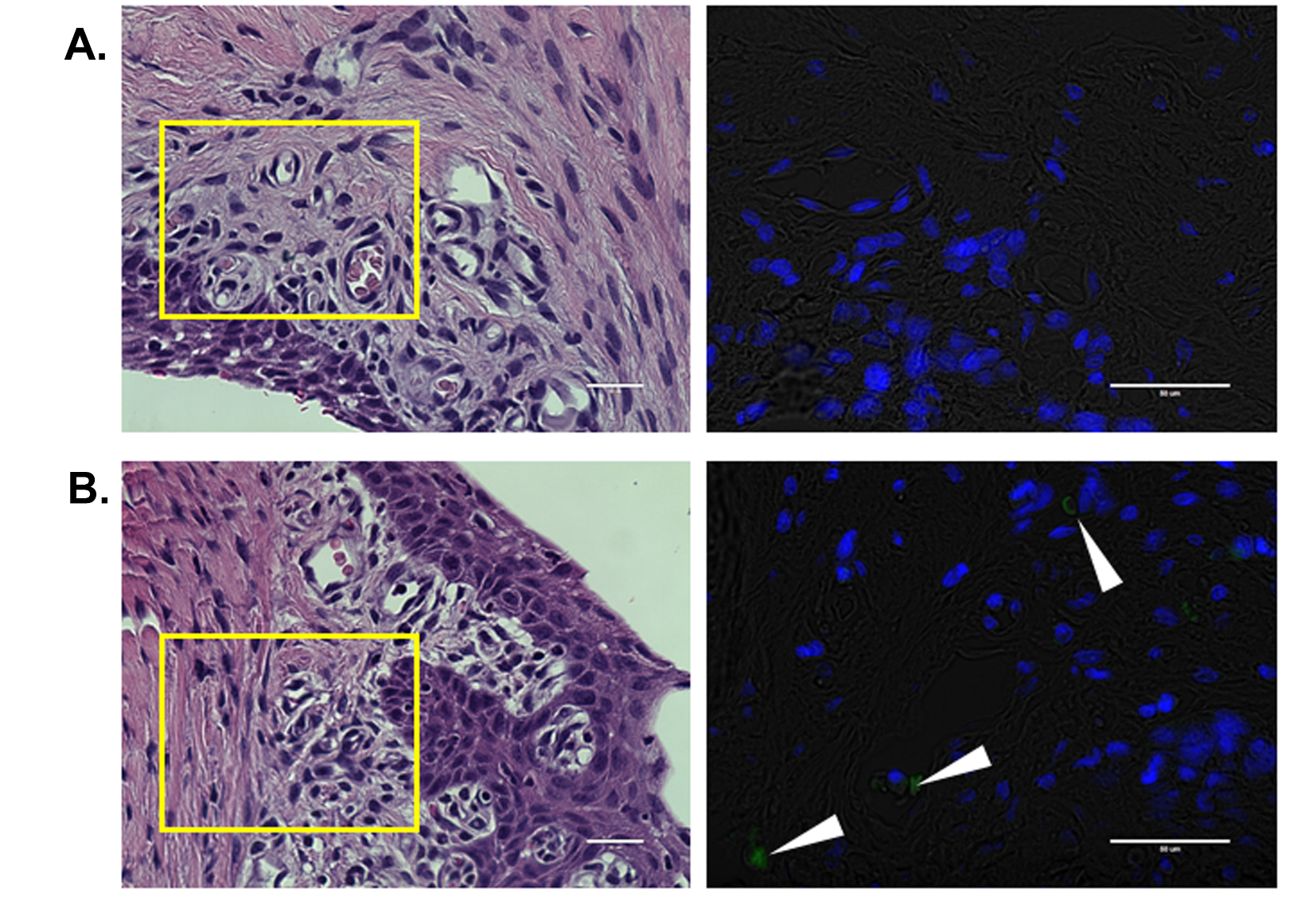


**Figure S2. *P. gingivalis* Immunostaining controls.** A) Representative images of immuno-fluorescent staining for *P. gingivalis* in a sham inoculated control. The corresponding H & E stained section on the left shows the approximate region (within the box) that is shown in the right panel. B) A representative image of the same specimen in Figure 2 that was stained with pre-immune serum (isotype control). The corresponding H & E stained section on the left shows the approximate region (within the box) that is shown in the right panel. Transillumination (grey) was used in immune-stained sections to demarcate tissue architecture and nuclei were stained with DAPI (blue). White arrowheads show autofluorescent red blood cells present in the section. Scale bars are equal to 50 μm.

**
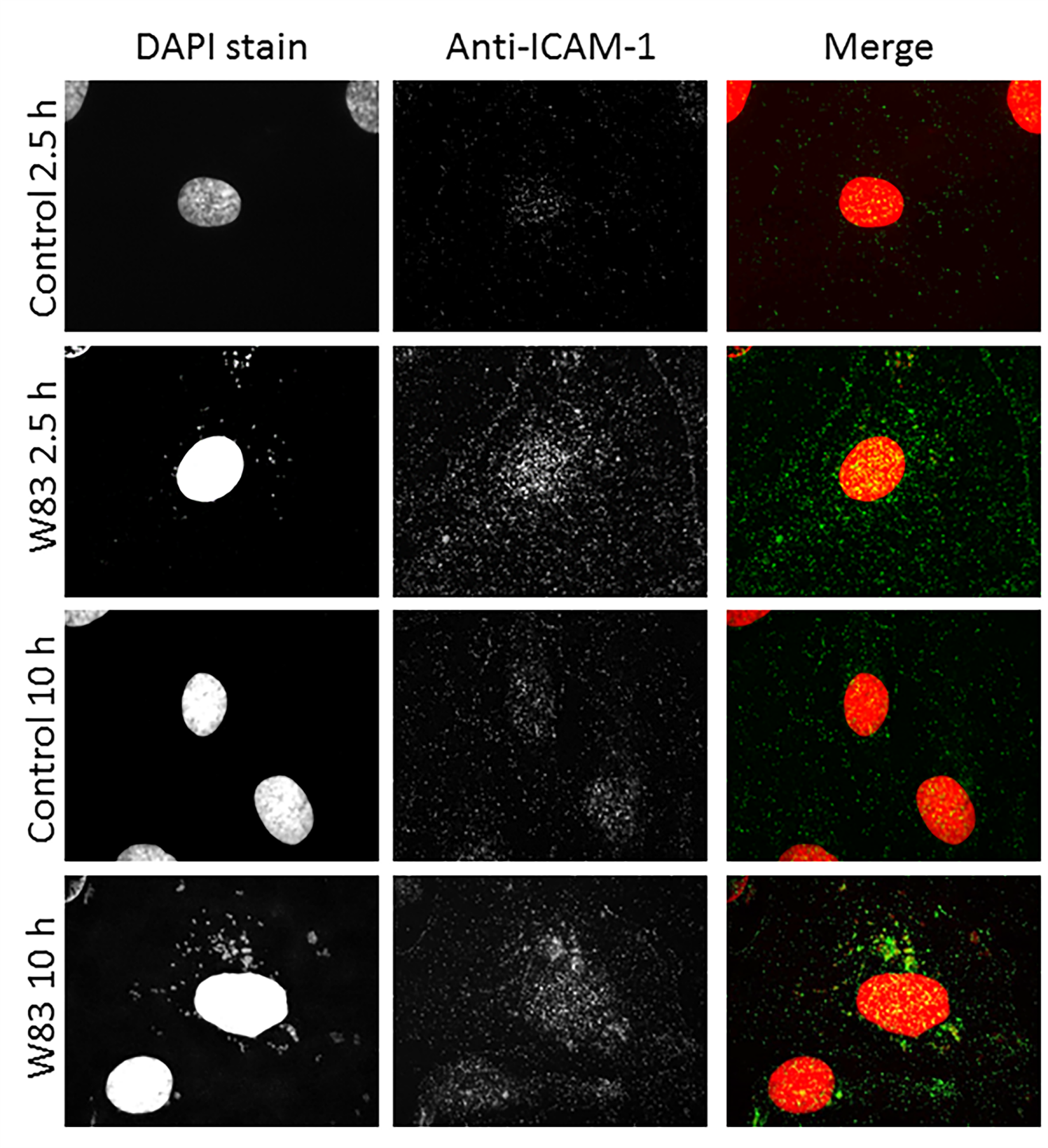
**

**Figure S3. Representative images of control and *P. gingivalis* inoculated HD-MVEC.** HD-MVEC were sham inoculated with sterile EnGS-MV medium or with a suspension containing *P. gingivalis* (MOI 100). At 2.5 and 10 h post-inoculation, cells were gently washed, and fixed before antibody labeling with anti-ICAM-1 antibody (pseudocolored green). Bacteria and host cell nuclei were stained with DAPI (pseudocolored red). Images are representative of 2 biological replicates.


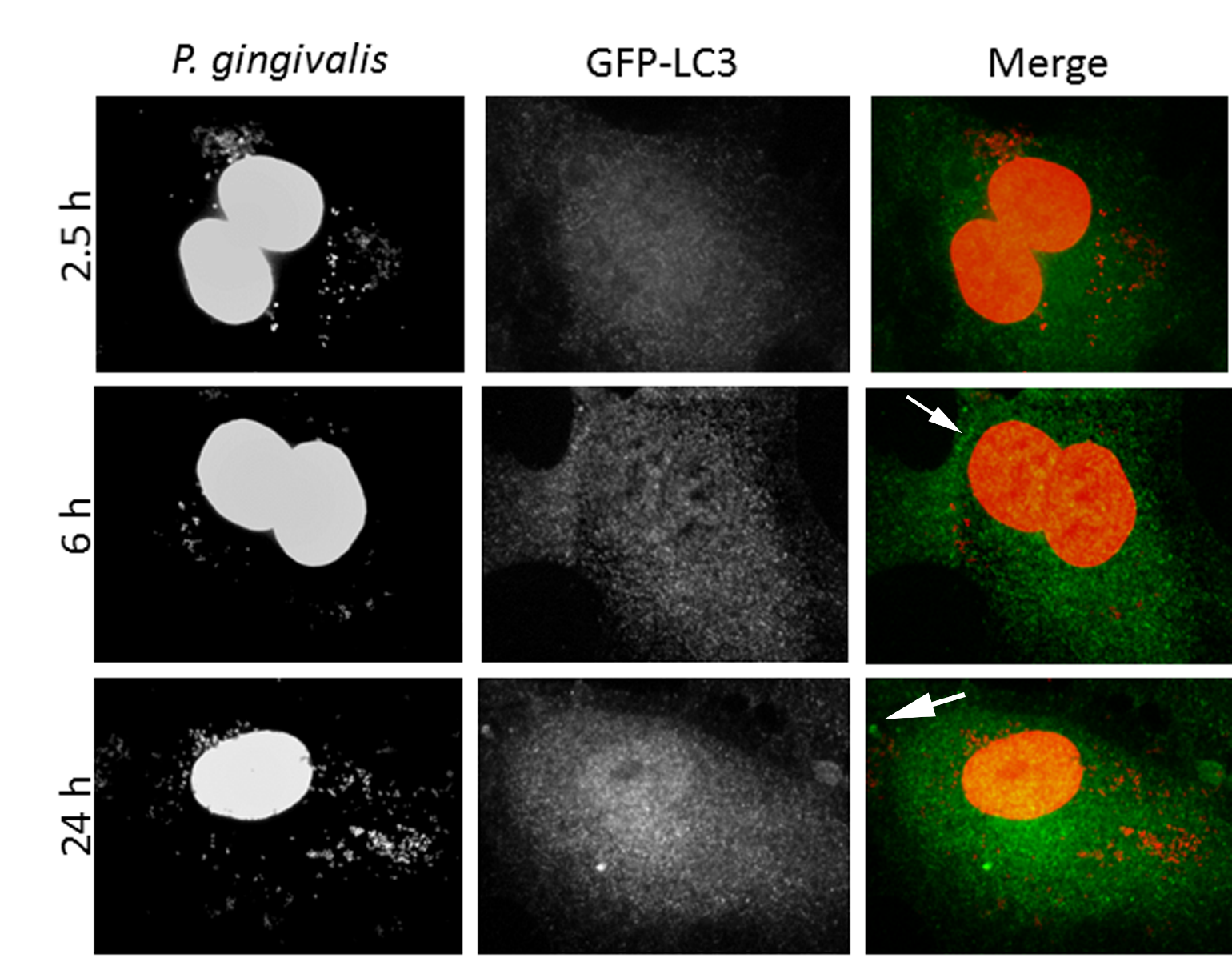


**Figure S4. Representative images of HD-MVEC transduced with GFP-LC3 adenovirus vector (Welgen, Inc, Worcester, MA, USA).** HD-MVEC were transduced 48 hours before infection with *P. gingivalis* strain W83 at an MOI of 100. GFP expression was checked by fluorescent microscopy at 24 h post-transduction (before infection with *P. gingivalis)*. Cells were harvested and processed at 2.5, 6, and 24 h post-inoculation. Images are representative of 20 to 30 cells from 3 biological replicates per time point.
